# Quality Assessment of the Clinical Practice Guidelines of Ostomy Care Based on the AGREE II Instrument

**DOI:** 10.3389/fpubh.2022.856325

**Published:** 2022-07-04

**Authors:** Xiaoyu Li, Qiao Yuan, Liangrong Geng, Zhiqi Chen, Rui Zhang, Liqun Guo, Shujin Yue

**Affiliations:** ^1^School of Nursing, Beijing University of Chinese Medicine, Beijing, China; ^2^Department of Preventive Treatment, Tianjin Hospital of ITCWM Nankai Hospital, Tianjin, China

**Keywords:** ostomy, guidelines, evidence, quality assessment, AGREE II

## Abstract

**Objectives:**

To assess the quality of clinical practice guidelines (CPGs) of ostomy care, and to analyze the status quo and challenges of guideline development.

**Methods:**

CPGs of ostomy care were systematically searched in relevant guideline websites and electronic databases, including PubMed, ProQuest, Web of Science, CNKI, VIP, WANFANG, and SinoMed, from January 1, 2012, to November 24, 2021. Two appraisers used the Appraisal of Guidelines for Research and Evaluation, 2nd edition (AGREE II) instrument to assess the quality of the included CPGs independently and objectively. The consistency of assessment was calculated using intraclass correlation coefficients (ICC).

**Results:**

A total of 5 CPGs relevant to ostomy care were assessed by AGREE II and the general quality of them was good. There were two CPGs of grade A and three CPGs of grade B. The domain scope and purpose (87.78%) had the highest scores, followed by the clarity of presentation (87.22%), the rigor of development (69.17%), stakeholder involvement (68.33%), and editorial independence (65.00%), and the lowest was applicability (55.42%). The overall assessment score was 5.40. All the ICCs for the AGREE II appraisal conducted by the two appraisers were >0.75.

**Conclusions:**

The five CPGs of ostomy care have the potential to be adopted in clinical practice. However, they still have some room for improvement, especially in the applicability domain. The development of ostomy care CPGs should follow the evidence-based progress and methodology of guideline formulation specifications while considering the effects of the CPGs and the practical issues.

## Introduction

An ostomy is a surgically created opening for the elimination of stool or urine that can be temporary or permanent (1). In developed countries, there is one stoma patient per 1,000 people. It has been reported that there are ~100,000 new permanent enterostomy patients in China every year, and the current cumulative number exceeds 1 million (2). As the incidence of intestinal and urinary malignancies continues to rise, it has significantly contributed to the increase in ostomy creation (3). A series of challenges after ostomy surgery, such as stoma complications (4), stoma maintenance (5), and body image issues (6) caused by changes in defecation or urination, can seriously affect the quality of life of patients with stoma. To improve the quality of life of patients after ostomy, many organizations or institutions have issued many clinical practice guidelines (CPGs). CPGs are tools that are developed to provide practical evidence for nursing. However, the quality of some CPGs of ostomy care is unknown. Recommendations from low-quality CPGs may mislead clinical nursing decision-making, causing damage and economic losses for ostomy patients and leading to the waste of limited medical resources at the same time (7, 8). A validated tool, named the Appraisal of Guidelines for Research and Evaluation, 2nd edition (AGREE II) instrument, was used to objectively assess the methodological quality of the included CPGs. This tool is now widely used to evaluate published scientific research and has been adopted by the World Health Organization Reproductive Health Library and other health care organizations to assess the quality of CPGs (9). This research aimed to use the AGREE II instrument to assess the quality of ostomy care CPGs published in the past 10 years and to analyze the state and challenges of guideline development.

## Methods

### Search Strategy

A systematic search related to primary and secondary publications was conducted in several journals that published CPGs. We searched the CPGs on ostomy care from the PubMed, ProQuest, Web of Science, CNKI, VIP, WANFANG, SinoMed, National Guideline Clearinghouse (NGC), National Institute for Health and Clinical Excellence (NICE), Scottish Intercollegiate Guidelines Network (SIGN), Guidelines International Network (GIN), Canadian Medical Association (CMA), New Zealand Guidelines Group (NZGG), Registered Nurses Association of Ontario (RNAO), Joanna Briggs Institute (JBI), National Health and Medical Research Council (NHMRC), Medlive, Chinese Nursing Association (CAN), Wound, Ostomy and Continence Nurses Society (WOCN), World Council of Enterostomal Therapists (WCET), and American Society of Colon and Rectal Surgeons (ASCRS) databases. The key words, including “ostomy,” “stoma,” “nursing,” “care,” “management,” “guideline,” “clinical practice guideline,” “guidance,” “recommendation”, “statement”, and “best practice,” were used to identify potentially eligible CPGs. We limited the language to English and Chinese. The published time limit was from “January 1, 2012, to November 24, 2021.” Taking PubMed as an example, the specific search strategy is shown in Table 1. In addition, the references from the retrieved studies were searched manually to avoid missing data.

**Table 1:** PubMed search strategy.

**Search strategy**
#1 (ostomy [MeSH Terms]) OR (stoma [Title/Abstract])
#2 (nursing [Title/Abstract]) OR (care [Title/Abstract]) OR (management [Title/Abstract])
#3 (guideline [Title/Abstract]) OR (clinical practice guideline [Title/Abstract]) OR (guidance [Title/Abstract]) OR (recommendation [Title/Abstract]) OR (statement [Title/Abstract]) OR (best practice [Title/Abstract])
#4 (“2012/01/01” [Date-Publication]: “2021/11/24” [Date-Publication])
#5 #1 AND #2 AND #3 AND #4

### Eligibility Criteria

The inclusion criteria were as follows: (a) evidence-based publications with clear and detailed documentation of the development methods; (b) publications that contained ostomy care and complication management-related content; (c) publications with complete guideline information, including the title, introduction, table of contents, contents, references and other details; (d) publications with revised or updated guidelines, included in the latest version; (e) publications published in either Chinese or English; and (f) publications published over the last 10 years (2012–2021). Guidelines were excluded if they met any of the following criteria: (a) were duplicated publications; (b) were guideline interpretations; (c) were guideline commentaries; (d) were reviews, consensus, and expert-based statements; (e) were not related to ostomy care, and involved only the diagnosis, medication, treatment and rehabilitation techniques of ostomy; (f) the topic was ostomy in children; and (g) the full text was unavailable.

### Literature Screening and Data Extraction

Duplications were identified and removed through reference management software (NoteExpress). The included studies were screened, extracted and double checked by two researchers independently. Disagreements were resolved by discussion or by consulting a third researcher. A data extraction table was designed using Excel software, and the information extracted from each article included the title, year of publication, country, organization, total number of references, and other factors.

### Quality Assessment of CPGs (AGREE II)

Using the AGREE II instrument, the quality of each CPG was independently assessed by two appraisers who were trained in the use principles and evaluation criteria of the instrument (10). When inconsistency existed among the assessors, discussions were conducted or a third researcher was consulted to reach an agreement.

The AGREE II instrument consists of 23 items organized in 6 domains, as well as two overall evaluation items. The six domains are the scope and purpose, stakeholder involvement, the rigor of development, the clarity of presentation, applicability, and editorial independence. Each item is scored on a scale from 1 (strongly disagree) to 7 (strongly agree) (10). The global score of each domain is calculated by summing the score of each item within the domain and then standardized via the following formula: (actual score—minimum possible score)/(maximum possible score—minimum possible score) × 100% (10). A higher percentage in the domains indicates that the CPGs are of higher quality (11).

On completing the 23 items, the appraisers provided an overall assessment of each CPG, which summarized the results of the six domains' scores and contained the personal judgments of the appraisers. On this basis, the CPGs were rated as grade A (strongly recommended) when the standardized percentages of all six domains were ≥60%, which can be directly recommended without any change; grade B (recommended with some modifications) when the standardized percentages ranged from 30 to 60% in more than 3 domains; and grade C (not recommended) when the standardized percentages were <30% in more than 3 domains (12).

### Data Analysis

IBM SPSS 20.0 software was used to examine the internal consistency (intraclass correlation coefficient, ICC) of the AGREE II quality assessment results of the two appraisers. The ICC value ranged from 0 to 1. It has been recommended that an ICC <0.4 indicates unsatisfactory consistency, an ICC ≥0.4 and <0.75 indicates generally acceptable consistency, and an ICC ≥0.75 indicates satisfactory consistency (13).

## Results

### Guideline Selection Process

**Figure 1:**
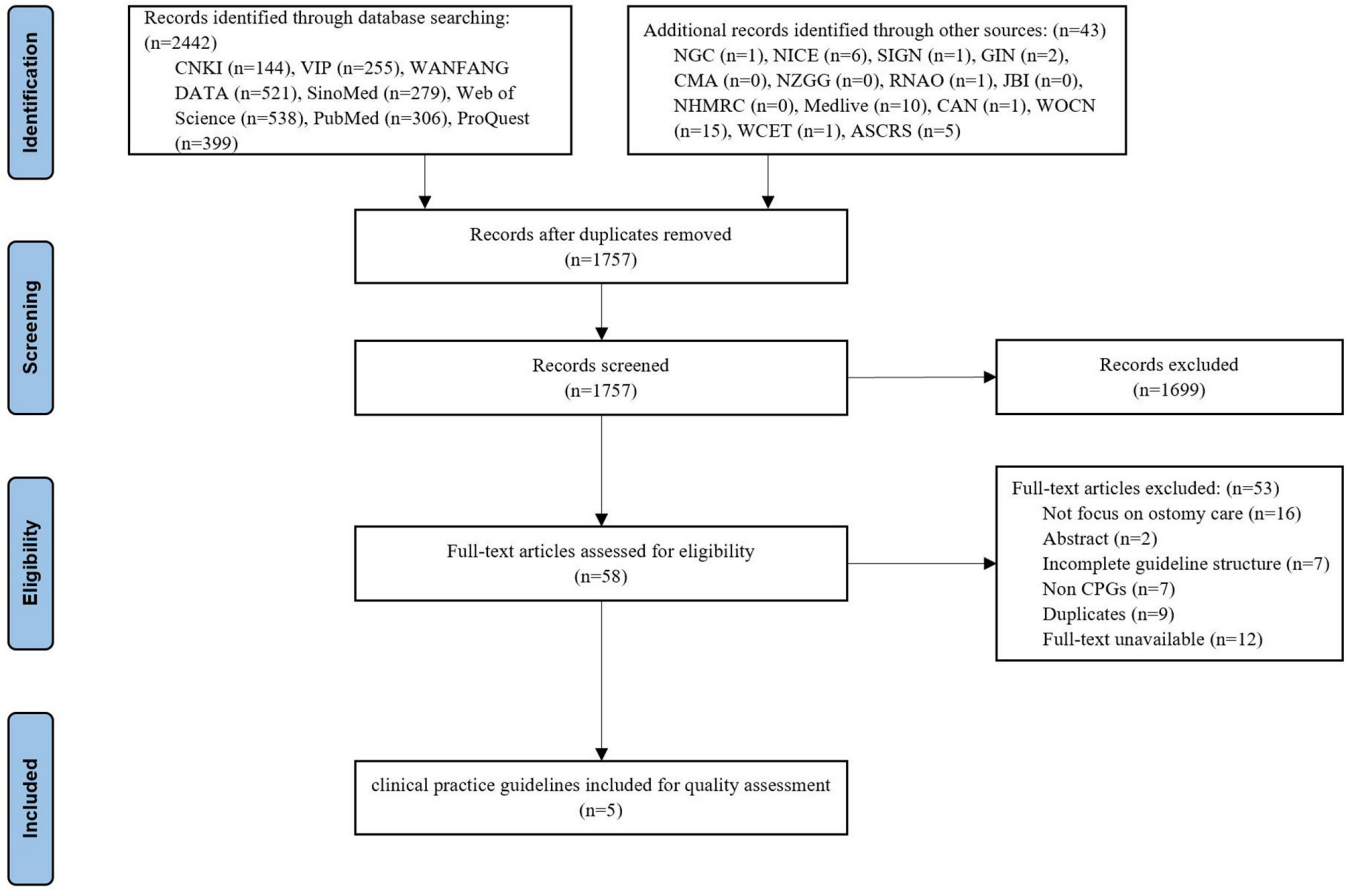
Flow diagram outlining the guideline selection process.

The initial search yielded 2,485 titles and abstracts, of which 728 were excluded as duplicates and 1,699 were removed after the abstracts were reviewed. Of the remaining 58 potentially eligible records, five CPGs (14–18) were finally included after reading the full text. The flow diagram of the guideline retrieval and selection process is shown in Figure 1.

### CPG Characteristics

A summary of the characteristics of the included CPGs is presented in Table 2. Five CPGs of ostomy care were assessed in our study, of which two were developed in the USA [one (17) developed by the Wound, Ostomy and Continence Nurses Society (WOCN Society) and the other (15) by the American Society of Colon and Rectal Surgeons (ASCRS)], two were developed in Canada [one (18) developed by the Ontario Provincial Enhanced Recovery After Surgery (ERAS) Enterostomal Therapy Nurse (ETN) Network and the other (16) by the Registered Nurses' Association of Ontario (RNAO)], and one (14) was developed in Italy [developed by the Multidisciplinary Italian Study group for STOmas (MISSTO)]. Four out of the five CPGs were issued or updated in the last 5 years.

**Table 2:** Characteristics of included CPGs.

**CPGs**	**Year of publication**	**Country**	**Organization**	**References**
Enhanced Recovery After Surgery: Best Practice Guideline for Care of Patients with a Fecal Diversion	2017	Canada	Ontario Provincial Enhanced Recovery After Surgery (ERAS) Enterostomal Therapy Nurse (ETN) Network	(18)
WOCN Society Clinical Guideline: Management of the Adult Patient with a Fecal or Urinary Ostomy	2017	United States	Wound, Ostomy and Continence Nurses Society (WOCN Society)	(17)
Clinical Practice Guidelines for Ostomy Surgery	2015	United States	American Society of Colon and Rectal Surgeons (ASCRS)	(15)
Italian guidelines for the surgical management of enteral stomas in adults	2019	Italy	Multidisciplinary Italian Study group for STOmas (MISSTO)	(14)
Supporting Adults Who Anticipate or Live with an Ostomy: Second Edition	2019	Canada	Registered Nurses' Association of Ontario (RNAO)	(16)

### Quality Assessment of Ostomy Care CPGs Based on the AGREE II Instrument

**Figure 2:**
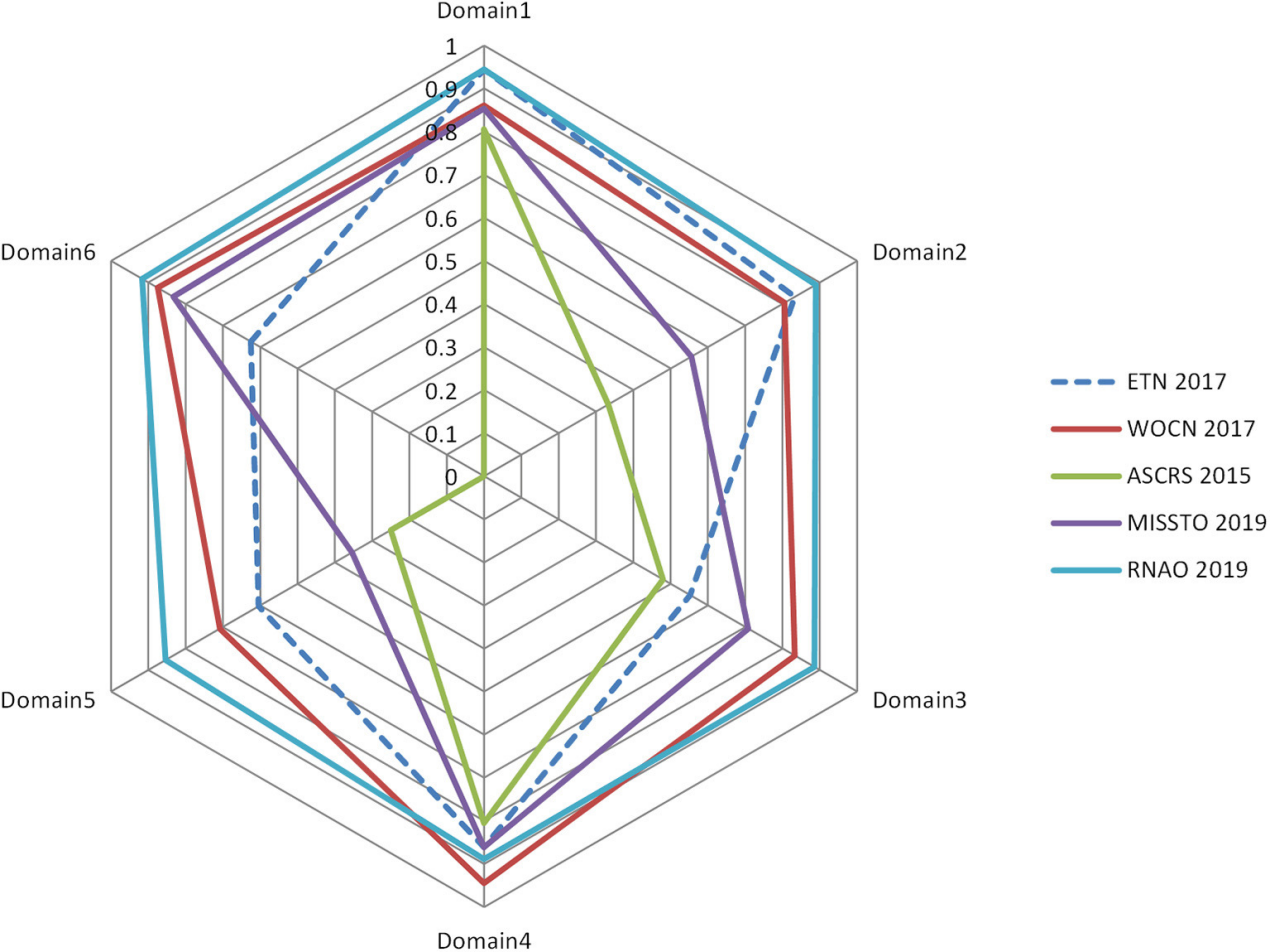
Radar map of the AGREE II final standardized domain scores for the five included ostomy guidelines.

Table 3 lists the standardized domain scores for each CPG in the six quality domains assessed with the AGREE II instrument. Of the 5 included CPGs, two (16, 17) were regarded as grade A, and three (14, 15, 18) were classified as grade B. The domain scope and purpose (median score of 87.78%) had the highest scores, followed by the clarity of presentation (median score of 87.22%), the rigor of development (median score of 69.17%), stakeholder involvement (median score of 68.33%), and editorial independence (medium score of 65.00%), and the lowest was applicability (median score of 55.42%). The overall assessment score was 5.40. All the ICCs for the AGREE II appraisal conducted by the two appraisers were >0.75, which indicated good coherence. The AGREE II final standardized domain scores for the five included ostomy guidelines are shown in Figure 2. Higher standardized domain scores map to the periphery. The graph visually shows the relative strength or weakness of each CPG by domain when compared to other included CPGs.

**Table 3:** Scores of the domains and overall assessment of the CPGs for ostomy care based on the AGREE II instrument.

**CPGs/AGREE II Domains**	**ETN 2017**	**WOCN 2017**	**ASCRS 2015**	**MISSTO 2019**	**RNAO 2019**	**M ±SD**
Domain 1. Scope and purpose (%)	94.44	86.11	80.56	83.33	94.44	87.78 ± 6.39
Domain 2. Stakeholder involvement (%)	83.33	80.56	33.33	55.56	88.89	68.33 ± 23.37
Domain 3. Rigor of development (%)	55.21	83.33	47.92	70.83	88.54	69.17 ± 17.50
Domain 4. Clarity of presentation (%)	86.11	94.44	80.56	86.11	88.89	87.22 ± 5.04
Domain 5. Applicability (%)	60.42	70.83	25.00	35.42	85.42	55.42 ± 24.94
Domain 6. Editorial independence (%)	62.50	87.50	0.00	83.33	91.67	65.00 ± 38.03
Overall assessment 1 (Overall quality)	4.50	6.00	4.50	5.50	6.50	5.40 ± 0.89
Overall assessment 2 (Recommend the CPG for use)	Yes with modifications	Yes	Yes with modifications	Yes with modifications	Yes	
Recommended grade	B	A	B	B	A	
ICC	0.800	0.793	0.920	0.858	0.776	

#### Domain 1: Scope and Purpose

This domain is an overall evaluation of the main purpose, health issues, and target population of the CPGs. The average score of the 5 CPGs was 87.78% [the lowest (15) was 80.56%, and the highest (16, 18) was 94.44%]. The results showed that each CPG had a clearly defined scope and purpose.

#### Domain 2: Stakeholder Involvement

This domain is a comprehensive evaluation of the staff who were involved in the development of the guidelines, which included specialists in ostomy and guideline development as well as patient representatives. The average score of the 5 CPGs in this domain was 68.33%, and 2 CPGs (14, 15) scored lower than the average, of which the lowest (15) was 33.33% as it only described the guideline developers only. Whether their recommendations represented the target population remains unknown.

#### Domain 3: Rigor of Development

This domain focuses on the evidence-based progress and methodology used to develop or update guidelines. The average score of the 5 CPGs in this domain was 69.17% [the lowest (15) was 47.92% and the highest (16) was 88.54%]. Two CPGs (15, 18) scored lower than the average as they had only a simple methodology presentation used to search evidence; in regard to the other evaluation items, they had little or no description. All the CPGs described the systematic method for searching evidence, but two CPGs (15, 18) had no description about the criteria for selecting evidence. All the authors took some health benefits, conflicts, side effects, and other risks into consideration while developing their recommendations. However, in most cases, the expression of the CPGs was inadequate or unclear. Four CPGs explicitly stated whether they were externally reviewed by experts prior to publication, while the remaining CPG (15) did not. Three CPGs (14, 15, 18) did not provide the relevant information about the way they updated the guidelines.

#### Domain 4: Clarity of Presentation

This domain addresses the language, structure, and presentation of the CPGs. The average score of the 5 CPGs in this domain was 87.22% [the lowest (15) was 80.56% and the highest (17) was 94.44%], which suggests that the 5 CPGs were generally well-reported. Each of the CPGs offered definite recommendations without any ambiguous advice. Furthermore, the key recommendations were very easy to identify by their emphasis in boldface, a flow chart, color in the conclusion list or other ways.

#### Domain 5: Applicability

The domain of application includes the evaluation of facilitators, barriers, and improvement strategies, as well as the relevant issues related to resources during the implementation process of the CPGs. The average score of the 5 CPGs in this domain was 55.42%; the lowest (15) was 25.00% and the highest (16) was 85.42%. Three CPGs (16–18) scored higher than the average. Three of the CPGs (15–17) discussed the obstacles and incentives for implementing recommendations but did not have detailed information and the other two (14, 18) contained no discussion about these topics. Four of them (15–18) provided tools or advice that helped support the implementation of the recommendations, and two CPGs (16, 18) had a clear description, which made their implementation easier. Although four CPGs (14, 16–18) mentioned the resource implications of implementing the recommendations, the information was not detailed. Four CPGs provided clear monitoring and auditing criteria, but the remaining CPG (15) contained little information on these criteria.

#### Domain 6: Editorial Independence

The domain of editorial independence aims to evaluate whether the implementation of the CPGs is influenced by the funding source and whether the competing interests of all the group members is stated. The average score of the 5 CPGs in this domain was 65.00%. The highest (16) was 91.67%, and the lowest (15) was 0% as it did not describe whether there were potential conflicts of interest.

## Discussion

Five clinical practice guidelines of ostomy care in 2012–2021 were assessed with the AGREE II instrument in consideration of regular updating of the guidelines. Most of the five CPGs were updated in the last 5 years, indicating that ostomy care is a matter of growing concern and has undergone certain development in recent years (19). The five CPGs are from three developed countries, the United States, Canada and Italy. The organizations are mainly academic institutions, such as the WOCN, ASCRS and RNAO, and most of them are non-profit organizations. The establishment of academic organizations makes the formulation of guidelines more standardized and feasible and promotes the vigorous development of the ostomy domain (20, 21).

The overall quality of the included CPGs was moderate to high. Two CPGs (40%) were rated as grade A, which means they can be recommended directly. Three CPGs (60%) were grade B, which can be recommended with modifications.

Among the five included ostomy care CPGs, the highest mean scores were achieved in the scope and purpose, as well as the clarity of presentation. The main weakness across the ostomy care CPGs was applicability. In the domain of scope and purpose, all the CPGs scored over 80%, which indicated that the main purpose, health issues, and target population of the CPGs were well-clarified. The clarity of the presentation domain received an average score of 87.22%, indicating that the health issues and recommendations in the five CPGs were well-presented and described and had a high degree of conformity with the guideline formulation specifications. Applicability is the key factor in the successful implementation of guidelines in clinical practice (22). The appraisal CPGs received the lowest scores in the applicability domain, indicating that the guideline developers did not pay sufficient attention to the potential barriers affecting the practical implementation of the recommendations, such as costs and hospital resources (23). To better facilitate the application of the guidelines, effective strategies such as guideline abstracts, flow charts, evaluation forms and other relevant resources could make it easier for the guidelines to be applied and implemented.

There are several strengths of our findings. On the one hand, this study used an international, rigorously structured and validated CPG assessment tool, AGREE II, to assess the quality of clinical practice guidelines for ostomy care, contributing to guide clinicians in the selection of high-quality, evidence-based guidelines in their daily practice. On the other hand, our authors have a research foundation in ostomy care and have received AGREE II training, and we have conducted a comprehensive search of databases and relevant websites, which ensured the reliability of the study results.

Inevitably, there were several potential limitations in this study. First, the review was limited to CPGs in Chinese or English, which may result in selection bias (21). Relevant CPGs intended for healthcare settings in other languages were excluded and may affect the generalizability of the results. Second, in view of the limited access to available foreign resources, in some cases, part of the full text could not be obtained, and some relative CPGs may have been missed by this research. Furthermore, the AGREE II instrument places emphasis on the methods of guideline development and the transparency of reporting but cannot assess the potential impacts of recommendations on patient outcome (24).

## Conclusion

In general, the five CPGs included in this study are of good quality, suggesting that they have the potential to be adopted in clinical practice. Despite this, the quality of these CPGs can be improved, especially in the domain of applicability. The development of ostomy care CPGs should follow the evidence-based progress and methodology of guideline formulation specifications while considering not only the effects of the CPGs but also some practical issues to improve the quality of ostomy care.

## Author Contributions

XL and SY conceived the study design. XL and QY searched and selected the articles. LGe and RZ assessed the quality of included guidelines. RZ and LGu extracted and analyzed the data. XL, QY, and ZC drafted the manuscript. SY revised the manuscript. All authors read and approved the final version of the manuscript.

## Funding

This research was supported by the Fundamental Research Funds for the Central Universities (2020-JYB-ZDGG-081).

## Conflict of Interest

The authors declare that the research was conducted in the absence of any commercial or financial relationships that could be construed as a potential conflict of interest.

## Publisher's Note

All claims expressed in this article are solely those of the authors and do not necessarily represent those of their affiliated organizations, or those of the publisher, the editors and the reviewers. Any product that may be evaluated in this article, or claim that may be made by its manufacturer, is not guaranteed or endorsed by the publisher.

## References

[1] Goldberg M Colwell J Burns S Carmel J Fellows J Hendren S. WOCN society clinical guideline: management of the adult patient with a fecal or urinary ostomyan executive summary. J Wound Ostomy Cont Nurs. (2018) 45:50–8. 10.1097/WON.0000000000000396 29300288

[2] Zhang W Luo Y Yao S Zhang J. Analysis of enterostomy research status and hotspots based on PubMed. J Nurs Sci. (2015) 30:85–9. 10.3870/j.issn.1001-4152.2015.24.085

[3] de Paula MA. [Performance of stoma therapy in the process of rehabilitation of ostomy patients]. Rev Bras Enferm. (1996) 49:17–22. 10.1590/S0034-71671996000100003 9052238

[4] Fellows J Voegeli D Hakan-Bloch J Herschend NO Storling Z. Multinational survey on living with an ostomy: prevalence and impact of peristomal skin complications. Br J Nurs. (2021) 30:S22–30. 10.12968/bjon.2021.30.16.S22 34514829

[5] Down G Vestergaard M Ajslev TA Boisen EB Nielsen LF. Perception of leakage: data from the Ostomy life study 2019. Br J Nurs. (2021) 30:S4–S12. 10.12968/bjon.2021.30.22.S4 34889680

[6] Mo J Wendel CS Sloan JA Sun V Hornbrook MC Grant M. Stoma location and ostomy-related quality of life among cancer survivors with ostomies: a pooled analysis. Am J Surg. (2021) 223:963–8. 10.1016/j.amjsurg.2021.09.023 34600739 PMC8948094

[7] Tang YC Shen G Hu SL Tang HQ Xu WP Xu TJ. Analysis and evaluation of China's clinical guidelines for management of chronic disease in primary clinics. Chinese J Hypertens. (2013) 21:48–52. 10.16439/j.cnki.1673-7245.2013.01.014

[8] Zhang F Kang X Wei B Wu H Li Z. Quality appraisal of clinical practice guidelines for acute heart failure using AGREE II. Chin Nurs Manag. (2016) 16:50–4.

[9] Brouwers MC Kho ME Browman GP Burgers JS Cluzeau F Feder G. AGREE II: advancing guideline development, reporting and evaluation in health care. Can Med Assoc J. (2010) 182:E839–42. 10.1503/cmaj.090449 20603348 PMC3001530

[10] Brouwers MC Kerkvliet K Spithoff K Consortium ANS. The AGREE reporting checklist: a tool to improve reporting of clinical practice guidelines. BMJ British Med J. (2016) 352:i1152. 10.1136/bmj.i1152 26957104 PMC5118873

[11] Yao L Chen Y Wang X Shi X Wang Y Guo T. Appraising the quality of clinical practice guidelines in traditional Chinese medicine using AGREE II instrument: A systematic review. Int J Clin Pract. (2017) 71:e12931. 10.1111/ijcp.12931 28382763

[12] Wang J Xu Y Chen Y Han L Jiang Y Zhao J. Quality appraisal of evidence-based guidelines on prevention and repair of perineal injury at vaginal delivery. Chin J Nurs. (2018) 53:162–8. 10.3761/j.issn.0254-1769.2018.02.007

[13] Koo TK Li MY. A guideline of selecting and reporting intraclass correlation coefficients for reliability research. J Chiropr Med. (2016) 15:155–63. 10.1016/j.jcm.2016.02.012 27330520 PMC4913118

[14] Ferrara F Parini D Bondurri A Veltri M Barbierato M Pata F. Italian guidelines for the surgical management of enteral stomas in adults. Tech Coloproctol. (2019) 23:1037–56. 10.1007/s10151-019-02099-3 31606801

[15] Hendren S Hammond K Glasgow SC Perry WB Buie WD Steele SR. Clinical practice guidelines for ostomy surgery. Dis Colon Rectum. (2015) 58:375–87. 10.1097/DCR.0000000000000347 25751793

[16] Registered Nurses' Association of Ontario (RNAO). Supporting Adults Who Anticipate or Live With an Ostomy. (2019). Available online at: https://rnao.ca/bpg/guidelines/ostomy (accessed November 24, 2021).

[17] Wound Ostomy Continence Nurses Society(WOCN). WOCN Society Clinical Guideline: Management of the Adult Patient With a Fecal or Urinary Ostomy. (2017). Available online at: http://www.wocn.org/ (accessed November 24, 2021). 10.1097/WON.0000000000000396 29300288

[18] Enterostomal Therapy Nurse Network(ETN),. Enhanced Recovery After Surgery: Best Practice Guideline for Care of Patients With a Fecal Diversion. (2017). Available online at: http://links.lww.com/JWOCN/A36 (accessed November 24, 2021).

[19] Vayssiere C Gaudineau A Attali L Bettahar K Eyraud S Faucher P. Elective abortion: clinical practice guidelines from the french college of gynecologists and obstetricians (CNGOF). Eur J Obstet Gynecol Reprod Biol. (2018) 222:95–101. 10.1016/j.ejogrb.2018.01.017 29408754

[20] Burch J. Care of patients undergoing stoma formation: what the nurse needs to know. Nurs Stand. (2017) 31:40–5. 10.7748/ns.2017.e10177 28589800

[21] Hazlewood GS Akhavan P Schieir O Marshall D Tomlinson G Bykerk V. Adding a “GRADE” to the quality appraisal of rheumatoid arthritis guidelines identifies limitations beyond AGREE-II. J Clin Epidemiol. (2014) 67:1274–85. 10.1016/j.jclinepi.2014.07.005 25240769

[22] Zhou H Deng L Wang T Chen J Jiang S Yang L. Clinical practice guidelines for the nutritional risk screening and assessment of cancer patients: a systematic quality appraisal using the AGREE II instrument. Supp Care Cancer. (2021) 29:2885–93. 10.1007/s00520-021-06094-z 33638747

[23] Husovich ME Zadro R Zoller-Neuner LL Vangheel G Anyangwe O Ryan DP. Process management framework: guidance to successful implementation of processes in clinical development. Ther Innov Regul Sci. (2019) 53:25–35. 10.1177/2168479018817751 30789099

[24] Watine J Friedberg B Nagy E Onody R Oosterhuis W Bunting PS. Conflict between guideline methodologic quality and recommendation validity: a potential problem for practitioners. Clin Chem. (2006) 52:65–72. 10.1373/clinchem.2005.056952 16391328

